# Acknowledgement to Reviewers of *Toxics* in 2018

**DOI:** 10.3390/toxics7010002

**Published:** 2019-01-09

**Authors:** Toxics Editorial Office

**Affiliations:** MDPI, St. Alban-Anlage 66, 4052 Basel, Switzerland

Rigorous peer-review is the corner-stone of high-quality academic publishing. The editorial team greatly appreciates the reviewers who contributed their knowledge and expertise to the journal’s editorial process over the past 12 months. In 2018, a total of 63 papers were published in the journal, with a median time to first decision of 19 days and a median time to publication of 43 days. The editors would like to express their sincere gratitude to the following reviewers for their cooperation and dedication in 2018:
Abbott, LouiseGuo, JingshuPalma, GiuseppeAguilar, CarmeHayashi, RyujiPaolo Busardó, FrancescoAlbano, EmanueleHewitt, Andrew JohnPappas, Richard StevenAlmeida, AgostinhoHImeno, SeiichiroParajuli, DurgaAncona, CarlaHöckner, MartinaParajuli, RajendraAnderson, Stacey E.Huertas-Pérez, José FernandoPattabiraman, MaheshAnifandis, GeorgeHung, Yung-TsePensieri, SaraAnticó, EnriquetaHursthouse, AndrewPeyton, David H.Arora, ManishHwang, Gi-WookPłotka-Wasylka, JustynaAvino, PasqualeHylander, Lars D.Pognonec, PhilippeBadot, Pierre-MarieIslam, M. NurulPovey, AndrewBalanay, Jo Anne G.Jenkins, Jill A.Puddu, Paolo EmilioBansal, Kuldeep K.Jovicic, VojislavRadford, Samantha A.Barst, BenjaminJulian, TimRagusa, Maria AntoniettaBharadwaj, LalitaKalinich, JohnRainieri, SandraBiegel-Engler, AnnegretKataoka, TomoyaRamaiahgari, Sreenivasa C.Brčić Karačonji, IrenaKatbamna, BhartiRegmi, BishnuBu, KaixuanKatsoyiannis, IoannisRice, Penelope A.Bustamante, PacoKern, JanetRico, AndreuCádiz-Gurrea, María LuzKlugbauer, NorbertRobinson, Stacey A.Candiani, SimonaKoch, WojciechRoca, MartaCaputo, Gregory A.Kudłak, BłażejRoig-Navarro, Antoni FrancescCarlsen, Hanne KrageLadeira, CarinaRolando, Carol A.Caserta, DonatellaLafforgue, MichelRubino, Federico MariaCaudle, MichaelLaforenza, UmbertoRusso, Mario VincenzoChappell, GraceLahooti, HooshangSá, RosáliaCheong, Yuen KiLarcombe, AlexanderSager, ManfredChiang, Yi-TingLauschke, VolkerSannagowdara, KumarChoi, KyunghoLavado, RamonSantilio, AngelaChyau, Charng-CherngLavkulich, Les M.Santos, JuanCiulu, MarcoLaw, DavidSaraiva, NunoColosio, ClaudioLee, Jin HeonSatoh, MasahikoConti, SaraLee, Jin-YongSavage, KayeCorreia, ManuelaLeondiadis, LeondiosScapellato, Maria LuisaCsavina, JanaeLi, AilinScheepers, Paul T. J.De Lourdes Gomes Pereira, MariaLiu, Hui-MingSchöpfer, JuttaDe Matteis, ValeriaLiu, KangzeSchreinemachers, DinaDe Simone, FrancescoLoudig, Olivier D.Shimek, Jo AnnaDeb, SubrataLucena, María IsabelSingh, Reetu R.Dekker, MarloesLutfy, KabirullahSogorb Sánchez, Miguel ÁngelDeLong, GayleM’kacher, RadhiaSolimini, RenataDeSesso, John M.Ma, YanSousa, Ana CatarinaDraheim, Roger R.Mansfield, EdwardStarostik, PetrDreij, KristianMargherita, MaioliStefanelli, PatriziaDrews-Botsch, CarolynMarion, Jason W.Stevenson, LouiseDunn, Ryan J. K.Marrs, James A.Su, ChunmingEbersviller, SethMartínez-Guitarte, José-luisSulheim, EinarEdwards, Joshua R.Mavrogonatou, EleniSuwazono, YasushiEl Muayed, MalekMcmorrow, TaraTagg, AlexanderElhaik, EranMcsorley, EmeirTakahashi, TsutomuErythropel, HannoMegalofonou, PersefoniTao, LimingEvens, AnneMeliker, JaymieTashiro, SatoshiExley, ChristopherMéndez, Loyda B.Teodoro, JoãoFay, Michael J.Miller, MarkThèvenod, FrankFord, DawnMitulovic, GoranThistle, HaroldFrias, JoãoMizukawa, HazukiTodorova, SvetoslavaFriedland, ReneMotta, Chiara MariaToft, GunnarGamble, Donald S.Moulis, Jean-MarcTsuji, JoyceGamet-Payrastre, LaurenceMurai, JunkoUlisse, SalvatoreGarcia, João Nuno Pinto MirandaMurkovic, MichaelVimercati, LuigiGardiner, PhilipMutter, JoachimWang, PingGay-Quéheillard, JeromeMyers, SandraWesterink, RemcoGebel, ThomasNaha, Pratap C.Williams, KatrynGeth, RichardNakai, KunihikoWilson, ThomasGhosh, AbhijitNees, MatthiasWoelwer-Rieck, UrsulaGillman, Ivan GeneNuñez, AlbertoYan, ShengminGinsberg, GaryODonnell, Greg E.Yasukazu, TakanezawaGoreham, ReneeOrdiales, Efrén García
Gramss, GerhardPagan, O. R.



